# Childhood post-adoption experiences and the long-term trajectories of deprivation-specific neurodevelopmental problems: a longitudinal study of the English and Romanian adoptee cohort

**DOI:** 10.1007/s00787-026-02968-x

**Published:** 2026-02-27

**Authors:** Maria Rodriguez-Perez, Mark Kennedy, Jana Kreppner, Edmund J.S. Sonuga-Barke

**Affiliations:** 1 Department of Child and Adolescent Psychiatry, Institute of Psychiatry, Psychology & Neuroscience, King’s College London, DeCrespigny Park, London, SE5 8AF UK; 2 Centre for Innovation in Mental Health, School of Psychology, University of Southampton, Southampton, UK; 3 Department of Child & Adolescent Psychiatry, Aarhus University, Aarhus, Denmark; 4 Department of Psychology, University of Hong Kong, Pok Fu Lam, Hong Kong

**Keywords:** Institutional deprivation, Autism, Professional intervention, Longitudinal study

## Abstract

Early institutional deprivation is associated with a co-occurring constellation of deprivation-specific problems (DSPs), including autism, ADHD, disinhibited social engagement (DSE), and cognitive impairment. While these difficulties often persist into young adulthood, substantial heterogeneity in developmental trajectories suggests that post-adoption environmental factors may influence recovery. This study adopts a transdiagnostic approach to examine how childhood experiences are associated with the long-term trajectories of these diverse neurodevelopmental and mental health domains. Using longitudinal data from 165 Romanian adoptees (total *N* = 217), we examined whether trajectories from ages 11 to young-adulthood for autism, ADHD, DSE, cognitive impairment, and mental health were predicted by childhood *negative family/peer experiences* and *professional intervention* (*clinical psychological therapies* and *special educational support*). We tested whether predictions differed between high versus low autism and ADHD symptom groups at age 11. Higher *Professional Intervention* levels were significantly associated with improving trajectories for autism, ADHD, DSE, and IQ, but not mental health outcomes. Multivariate analysis suggested complementary roles of *clinical psychological therapies* and *special educational support*; the former being more strongly associated with ADHD and DSE, and the latter with autism and IQ. Associations were observed across the symptom severity continuum for autism and ADHD, with comparable effect sizes found in both high and low symptom groups. *Negative family and peer experiences* showed some bivariate associations but were not significant predictors in multivariate models. Childhood *professional intervention* was associated with improved deprivation-specific outcomes, with effects across symptom severity levels, highlighting tractability despite strong persistence. The differential and complementary effects suggest comprehensive support requires both educational and clinical interventions.

**Supplementary Information:**

The online version contains supplementary material available at 10.1007/s00787-026-02968-x.

## Introduction

Autism is a lifelong neurodevelopmental condition that is clinically characterised by persistent difficulties with communication and social interaction, restricted, repetitive and stereotyped behaviours, interests and/or activities, and sensory anomalies [1]. While the condition typically has strong genetic underpinnings, as suggested by twin and molecular studies [2], its aetiology is understood to be multifactorial, with various environmental factors, including a range of pre-conception (e.g., parental age and maternal obesity [3–5]), prenatal (e.g., experiences to viral and bacterial infections, toxins, and maternal stress [6–9], and perinatal influences (e.g., prematurity, pregnancy and labour and delivery complications [10–13].

In contrast, little evidence exists for postnatal environmental influences on autism. One exception to this comes from the English and Romanian Adoptees (ERA) study of the effects of institutional deprivation on development. In this study, adoptees who had been exposed to more than six months of extreme deprivation in the Romanian institutions of the late 1980 s were at an elevated risk of displaying a range of overlapping neurodevelopmental difficulties compared to those exposed to less than 6 months of deprivation. These included attention-deficit/hyperactivity disorder (ADHD), disinhibited social engagement (DSE), and cognitive impairment (i.e., IQ < 80). Perhaps most striking was the high rates of marked autistic features in this group. Twenty children were identified at age 4/6 [14], as meeting clinical threshold on the *Autism Diagnostic Interview* (ADI-R [15]. A further eight children were identified at age 11/12 [16] as meeting a cutoff of over 14 on the total score of the *Social Communication Questionnaire* (SCQ; see Rutter et al. [16], for rationale). This group were initially dubbed as *quasi-autistic* (QA; [14]) because, compared to typical autistic individuals in clinics, their autistic features had a putatively non-genetic basis, seemed to be resolving clinically during development and had an unusual clinical presentation (e.g., accompanied by an unusually high level of social approach – linked to its overlap with DSE).

Given this designation, it was somewhat surprising to discover from longer-term longitudinal studies of this cohort that autism symptoms were in fact remarkably persistent across adolescence [16] and into young-adulthood [17], despite enrichment in typically high-functioning and well-resourced adoptive families. Furthermore, in our recent young-adult follow-up of the QA group specifically, we found that this marked persistence was seen across all three core autism domains (communication, social reciprocity and repetitive and stereotyped behaviours), strongly overlapped with other deprivation-related neurodevelopmental problems (e.g., ADHD) and was associated with a profound impact on daily functioning and mental health [18]. Importantly, autism symptoms were also present in the subclinical range among Romanian adoptees who did not meet the QA threshold but experienced over six months of deprivation. This suggests that while the QA phenotype represents the most severe manifestation, the developmental impact on autism domains exists as a dimensional continuum of risk across the entire group exposed to prolonged institutionalisation. In a companion paper, we compared the QA profiles and outcomes with an early community diagnosed autism sample [19]. We found a significant degree of similarity in the overall autism profile between deprived and community autistic individuals, despite some distinctive characteristics. For example, within the QA group, the communication domain mainly comprised persistent abnormalities of linguistic expression, whereas difficulties with social reciprocity, while present, were somewhat less severe and tended to decline over time. The outcomes observed in the ERA cohort are largely consistent with patterns reported in other international studies of children adopted from similarly deprived institutional settings. Specifically, the presence of autism symptoms [20, 21], as well as high rates of persistent ADHD and cognitive impairment [22–26], mirror findings from cohorts in the US, Canada, and across Europe.

Despite the overall persistence of autism symptoms into young-adulthood in the ERA cohort, along with associated daily functional and mental health difficulties, in a substantial minority of the adoptees with prolonged early deprivation experience, there was also considerable within-group variability. Some individuals showed greater persistence of difficulties than others. Understanding the factors that contributed to this variability may represent a first step in practical attempts to identify tractable factors that could provide intervention targets to improve outcomes in deprived or otherwise maltreated children. Additionally, while DSPs often co-occur, it remains unclear whether they follow shared or distinct recovery trajectories. Accordingly, the current paper adopts a transdiagnostic approach to explore the broad pattern of associations between post-adoption experiences and trajectories of neurodevelopmental and mental health difficulties across adolescence and into early adulthood. We were interested in both putatively-positive (i.e., *professional intervention*) and -negative experiences (i.e., *negative family and peer-related experiences)*.

We examined the influence of *professional intervention* in terms of degree of engagement with *clinical psychological therapies* and *special educational support* received by the adoptees during childhood. It is notable, though perhaps not surprising, that nearly one-third of children exposed to extended periods of deprivation had received mental health services by age 11 [27], with service provision being strongly linked to the *deprivation specific problems* mentioned above (DSPs; i.e., QA, ADHD, DSE [28]). *Special educational provision* was also commonly accessed by age 11, with almost 40% of children exposed to over 6 months of deprivation receiving substantial special educational support, including special schooling, formal assessments, or being held-back a year [27]. Previous research on the long-term effectiveness of *psychological interventions* for autism presents a complex picture. While many psychoeducational and clinical approaches are considered evidence-based and first-line, findings regarding their long-term impact on symptom trajectories remain varied [29–31]. Some longitudinal studies and meta-analyses suggest that early support is associated with gains in adaptive behaviour and cognitive ability [29, 32, 33]. However, the persistence of these improvements into adulthood is not always clear, and effect sizes often vary depending on the methodological rigor of the studies and the specific developmental domains targeted [31, 32, 34]. Overall, the substantial variability in participant characteristics and intervention approaches across different studies make definitive conclusions regarding long-term efficacy challenging, which underscores the importance of identifying which developmental trajectories are most strongly associated with childhood support within specific neurodevelopmental profiles, such as those associated with early institutional deprivation.

We also had available data on *negative family and peer experiences* in childhood (including bullying). There is little evidence to date from the ERA study that adoptive family characteristics are influential in determining outcomes associated with deprivation – probably because the families were generally well-functioning, with minimal significant ‘issues’ being reported [35]. Peer relationships, particularly bullying, were not directly linked to emotional difficulties at age 11 [35]. However, later work by Rizeq et al. [36] identified a pathway from early experiences-of-deprivation-to-poor young-adult mental health, mediated by early neurodevelopmental problems and later bullying experiences, using cross-lagged analysis. More generally, while maternal mental health may impact child behaviour [37], its role in predicting autism symptom trajectories is less clear [38]. Bullying victimisation, while not directly altering autism symptom trajectories, significantly influences mental health risks in autistic individuals [39], which may indirectly affect social communication [40]. Similarly, the experience of adverse life events is a recognised risk factor for co-occurring mental health issues in autistic individuals [41, 42], potentially shaping important life-experiences and functional outcomes even if not directly altering core symptom trajectories [2, 43].

In the current study we asked four questions:

1. Are *negative family and peer experiences* and/or *professional intervention* in childhood associated with long-term neurodevelopmental trajectories -including autism, ADHD, DSE, and cognitive impairment – as well as young-adult, mental health outcomes?

Throughout this paper, we use the term “improving trajectory” to refer to favourable autism, ADHD, DSE, and mental health outcomes (i.e., greater symptom reduction over time), which statistically corresponds to negative slopes for symptom severity measures. Conversely, “deteriorating trajectory” for these outcomes refers to unfavourable outcomes (i.e., increase/worsening of symptoms over time), corresponding to positive slopes. For IQ scores, the direction is reversed: improving trajectories correspond to positive slopes (increasing IQ scores), while deteriorating trajectories correspond to negative slopes (decreasing IQ).

Based on these definitions, we hypothesise that higher levels of *professional intervention* and lower levels of *negative family and peer experiences* would be associated with improving trajectories across all neurodevelopmental and mental health domains – highlighting the potentially tractable nature of deprivation-related problems despite their high levels of general persistence.

2. What is the relative importance of *clinical psychological therapies* and *special educational support* in childhood in their association with trajectories of autism, ADHD, DSE, cognitive impairment, and mental health, and do these associations reflect domain-specific or transdiagnostic patterns of recovery?

We hypothesise that both intervention types would show significant associations with improving trajectories, given the wide range of difficulties experienced by the ERA cohort. However, mental health outcomes would be more strongly associated with clinical interventions.

3. Do the associations between childhood predictors and young-adult outcomes vary based on baseline symptom severity (comparing high- vs. low-symptom groups)?

We hypothesised that the predictive power of both *professional interventions* and *negative family and peer experiences* would be stronger for all outcome trajectories in the group with high levels of baseline (i.e., age 11) autism symptoms.

4. Are the differential effects for high vs. low symptom levels on predictions specific to autism or do they generalise to ADHD?

We hypothesised that these differential predictions will also be seen in comparison of high vs. low ADHD, possibly reflecting an element of shared underlying vulnerability associated with deprivation-related autism and ADHD.

## Methods

### Participants

The full ERA sample is described in detail in earlier papers [17]. One hundred and sixty-five Romanian adoptees (91 female, 55%) and their families entered the *English & Romanian Adoptees* (ERA) study in the mid-1990s. Romanian adoptees typically entered institutions in the first few months of life and experienced between 1-and-43 months of extreme global deprivation while living in Romanian orphanages (average length of deprivation 12.5 months – Table 1). ERA study retention was high up to age 15 years (data available from 90% of the sample) but decreased into young-adulthood (data missing for 25% of the sample). There was no evidence of selective attrition comparing those dropping out prior to the young-adult assessment and those who remained in the study [17] (data available on request). The study was approved by the ethics committees at King’s College, London, UK (5447) and the University of Southampton, UK (14308).

**Table 1:** Summary of characteristics at age 11, childhood predictors and young-adult outcomes for the whole sample and subgroups

Background characteristics	Whole sample (*N* = 165)	Low Autism (*n* = 70)	High Autism (*n* = 80)	Test	Group comparison
**Exact age at age 11 assessment**,** mean (SD)**	11.15 (0.27)	11.12 (0.12)	11.19 (0.19)	**t (128.97) = −2.69**, ***p =.008***	**High Autism > Low Autism**
**Sex**,** female (n**,** %)**	91 (55.2)	37 (52.9)	43 (53.8)	$\:{X}^{2}$(1) = 0.001, *p* =.55	-
**Duration of deprivation (months)**,** mean (SD)**	12.47 (11.36)	9.53 (10.80)	14.90 (11.39)	t (148) = −2.95, *p* =.004	**High Autism > Low Autism**
**Age 11 DSPs and Emotional problems**					
**Autism**,** mean (SD)**	4.02 (4.29)	1.61 (1.17)	7.82 (3.90)	**t (94.90) = −13.40**, ***p <.001***	**High Autism > Low Autism**
**DSE**,** mean (SD)**	0. 74 (1.09)	0.50 (0.94)	0.94 (1.16)	**t (144.94) = −2.49**, ***p =.01***	**High Autism > Low Autism**
**ADHD**,** mean (SD)**	0.61 (0.95)	0.29 (0.68)	0.89 (1.07)	**t (134.01) = −4.12**, ***p <.001***	**High Autism > Low Autism**
**IQ**,** mean (SD)**	90.29 (19.44)	95.68 (19.75)	85.19 (17.89)	**t (142) = 3.14**, ***p <.001***	**High Autism < Low Autism**
**Emotional problems**,** mean (SD)**	0.67 (0.50)	0.61 (0.50)	0.73 (0.51)	t (138) = −1.40, *p* =.16	-
**Childhood experiences**					
**Has been bullied**,** n (%)**	69 (52.3)	32 (56.1)	34 (50.7)	$\:{X}^{2}$(1) = 0.36, *p* =.34	**-**
**Adverse life events**,** mean (SD)**	4.42 (2.83)	3.78 (2.57)	4.91 (3.02)	**t (122) = −2.21**, ***p =.03***	**High Autism > Low Autism**
**Mother mental health**,** mean (SD)**	3.30 (2.90)	2.78 (2.49)	3.77 (3.16)	**t (137) = −2.03**, ***p =.04***	**High Autism > Low Autism**
**Clinical psychological therapies**,** n (%)**	44 (26.7)	10 (14.3)	32 (40.1)	$\:{\boldsymbol{X}}^{2}$**(1) = 12.26**, ***p =.002***	**High Autism > Low Autism**
**Special educational support**,** n (%)**	74 (44.8)	25 (35.7)	44 (55)	$\:{\boldsymbol{X}}^{2}$**(1) = 5.59**, ***p =.01***	**High Autism > Low Autism**
**Any form of intervention**,** n (%)**	83 (0.3)	27 (38.6)	51 (63.7)	$\:{\boldsymbol{X}}^{2}$**(1) = 9.48**, ***p =.002***	**High Autism > Low Autism**
**Developmental trajectories/outcomes**					
**Autism Slope**	−0.17 (0.76)	0.20 (0.42)	−0.50 (0.87)	**t (114.67) = 6.40**, ***p <.001***	**High Autism < Low Autism**
**DSE Slope**	−0.11 (0.14)	−0.08 (0.12)	−0.13 (0.15)	**t (137.01) = 2.84**, ***p =.003***	**High Autism > Low Autism**
**ADHD Slope**	0.04 (0.40)	0.13 (0.30)	− 0.04 (0.47)	**t (143.99) = 3.66**, ***p <.001***	**High Autism < Low Autism**
**IQ Slope**	2.29 (5.29)	1.57 (5.21)	2.81 (5.53)	t (144.98) = 0.37, *p* =.14	**-**
**Mental Health**	0.16 (1.04)	− 0.17 (0.86)	0. 52 (1.11)	**t (88.80) = −3.41**, ***p <.001***	**High Autism > Low Autism**

The sample includes Romanian adoptees only (*N* = 165). Figures in **bold** indicate significant effects (i.e., *p* <.05)

*SD* Standard deviation. *DSE* Disinhibited Social Engagement. *ADHD* Attention Deficit Hyperactivity Disorder. *DSPs* Deprivation specific problems

### Measures

#### Background characteristics

Data on Romanian adoptees’ sex, date of birth, and duration of institutional deprivation were obtained from Romanian records collected at the time of entry into the United Kingdom (UK). Duration of deprivation (in months) was calculated based on the adoptees’ age at UK entry.

## Outcomes

### Autism symptoms

An adapted version of the *current* form of the Social Communication Questionnaire (SCQ; [44]), a clinically validated parent-report screening test for autism symptomatology that maps onto the three DSM-IV [45] diagnostic criteria, was used to assess autism symptoms at ages 11-, 15- and 23-years for the ERA groups. The SCQ has high internal consistency (Cronbach’s alpha = 0.87; [44]), and good discriminative validity when distinguishing between children with autism and non-autism at all IQ levels, particularly when administered in children 4 years of age or older [46]. The original SCQ consists of 40 items. However, for our analysis we selected five items from each domain which were developmentally appropriate across all assessment waves. These can be seen in Appendix I (see [17] for the rationale for this approach). Items were rated 0 for “absent” or 1 for “present”. Reverse scoring (i.e., recoding responses so that the numerical scoring scales runs in the opposite direction) was performed when relevant, so that a score of 1 would always reflect a positive symptom endorsement. The SCQ total score was calculated as the sum of individual items.

### Disinhibited social engagement

Symptoms of DSE were assessed at ages 11, 15, and in young-adulthood using researcher ratings of parents’ answers to three interview questions. These questions focused on behaviours such as being overly friendly, showing inappropriate intrusiveness and being unaware of social boundaries. A rating of “definitive evidence of disinhibition” (rating of 2 on a 0–2 scale) represented a positive endorsement.

### Attention-deficit/hyperactivity disorder

To ensure developmental consistency across assessment waves, hyperactivity, sustained attention and distractibility were measured using equivalent items from the Revised Rutter Scale (Elander & Rutter, 1996) at age 11, the Strengths and Difficulties Questionnaire (SDQ; [47]) at age 15, and the Conners Comprehensive Behaviour Rating Scale (CBRS; [48]) in young-adulthood. For the Revised Rutter Scale and the SDQ, a symptom was deemed endorsed when a rating of 2 (‘certainly applies’) was made (0–2 scales). The equivalent rating (‘often/very often’, rating of 2 or 3) was found in the CBRS (0–3 scale; see [17] for rationale).

### IQ

Cognitive functioning was assessed using the short form (i.e., block design, objects assembly, vocabulary, and similarities) of the Wechsler Intelligence Scale for Children (WISC-III^UK^ [49]), at ages 11 and 15. In young-adulthood, the block design and vocabulary subscales from the short-form Wechsler Abbreviated Scale of Intelligence (WASI) [50] were used.

### Mental health

Symptoms of several mental health conditions– including generalised anxiety, major depression, oppositional defiant disorder, manic episode, social phobia, conduct disorder and obsessive-compulsive disorder– were assessed through parental report using the Conners Comprehensive Behaviour Rating Scales (Conners et al., 2011) using T-scores on a 0–3 scale. Parental report was chosen over self-report due to higher completion rates by parents. However, it’s noteworthy that previous findings by Sonuga-Barke et al. [17] showed comparable levels of elevated emotional problems reported by both parents and adoptees in young-adulthood within the High Deprivation group (i.e., > 6 months of deprivation).

### Predictors

#### Professional intervention in childhood

##### Clinical psychological therapies

Parents were interviewed during the age 11 assessment to gather information about their child’s lifetime history of psychological support up until that age. Clinical psychological therapy provision was defined in terms of at least a two-session contact with a mental health professional that led to either a psychiatric diagnosis or the conclusion that a clinically significant disorder was present, though potentially undiagnosed (see Castle et al., 2006). The answers received binary coding, where 1 indicated the child had received psychological support and 0 indicated the absence of it. As part of the study, families were offered the opportunity to consult with Michael Ruter (MR), a consultant psychiatrist, for diagnostic evaluation and guidance on accessing local services, though ongoing treatment was not provided through the study. While 28 families accessed this consultation, it was only coded as 1 (i.e., received psychological support) if accompanied by external mental health professional contact, except in four cases where MR provided two or more direct clinical sessions.

##### Special educational support

Parents responded either “yes” (coded as 1) or “no” (coded as 0) to whether their child received special support in school during interviews conducted as part of the age 11 assessment. Special educational support was categorised as present if, up to the age of 11 years, the child had attended a special school, had received an individual education plan in a state school or equivalent in private school, or had been held back a year (see [27]).

#### Negative family and peer experiences in childhood

##### Maternal mental health

The Malaise Inventory [51] was administered to adoptive mothers during the age 11 assessment. This is a 24-item questionnaire covering emotional and somatic symptoms intended to measure psychological distress. The Malaise Inventory was derived from the Cornell Medical Index Health Questionnaire, which was comprised of 195 self-completion items [52]. Items were scored binary (0 = No/1 = Yes). Individuals responding ‘yes’ to eight or more of the 24 items are considered to be at risk of depression [53]. The internal consistency of the 24-item scale has been found to be acceptable [53, 54]. A mean score was calculated for this study.

##### Bullying victimisation

We assessed bullying victimisation using parents’ responses to one interview question at age 11 (“ever been bullied”). Responses were coded binary, with 1 for “any report of bullying” and 0 for “no indication of bullying.”

##### Adverse life events

Experience of negative life events between ages 11 and 15 was assessed with a Life Event Scale that consists of 30 questions, answered using binary coding (0 for “no”, 1 for “yes”). The scale was adapted from the Johnson Scale [55]. A sum score was created for the current analyses.

### Statistical analyses

#### Preparatory analyses

##### Predictor variables

Before answering our research questions, we first explored the correlations between predictor variables within each domain: *negative family and peer experiences*, and *professional intervention* (see Methods for specific variables under each domain). This analysis tested whether variables within each domain were sufficiently correlated to justify the application of Principal Component Analysis (PCA) to reduce the number of predictor and outcome variables. The correlation analyses supported the use of PCA (see Appendix II – Table 3 for correlations) to reduce the number of statistical tests conducted and ease the interpretation of findings. Based on this we created unweighted composite scores that preserved the conceptual coherence identified through PCA while maximising data retention. Specifically, for each participant, we calculated composite scores using an available-case approach: individual item scores were Z-transformed, then averaged across all available variables within that domain for each participant. This approach uses all available data rather than excluding participants with any missing values, under the assumption that data are missing at random and that available variables provide a representative estimate of each participant’s standing on the underlying construct [56, 57].

##### Outcome variables

Our outcomes of interest were changes in symptoms (i.e., deterioration or improvement from age 11 through to young-adulthood). To create these variables for autism symptoms, ADHD, DSE, and cognitive impairment (i.e., IQ < 80 points), while minimising the impact of missing data on statistical power, we used Latent Growth Models (LGM). These models estimated developmental trajectories as linear slopes representing symptom change from age 11 through age 15 to young-adulthood (average age 23.5 years). For the *mental health outcomes in young-adulthood*, however, we judged that a developmental slope could not be derived because, although assessed longitudinally across all ERA assessment points, the measures used at different time points differed too much from each other. Therefore, our analysis of change in mental health outcomes involved examining predictions of young-adult outcomes (average 23.5 years) while controlling for childhood (age 11) emotional problems (Table 1), measured by the Revised Rutter Scale [58], and entered as a covariate in partial correlation analyses.

## Research Question 1: Are *negative family and peer experiences* and/or *professional intervention* in childhood associated with long-term neurodevelopmental trajectories -including autism, ADHD, DSE, and cognitive impairment – as well as young-adult, mental health outcomes?

First, we conducted bivariate Pearson correlation analyses between predictors and outcome trajectory slopes (as noted earlier in the Introduction, positive slopes statistically indicate deteriorating trajectories, except for IQ where positive slopes indicate improving trajectories). Second, to isolate the independent significance of each predictor, we conducted a series of multiple linear regression analyses for each outcome. We entered both predictors simultaneously as well as background characteristics (i.e., sex, exact age at 11, and duration of deprivation). For the model predicting mental health outcomes, we added additional covariates mirroring the approach used in our partial correlations, where we also controlled for baseline emotional problems and the effect of the other DSPs at age 11.

## Research Question 2: What is the relative importance of clinical psychological therapies and special educational support in childhood in their association with trajectories of autism, ADHD, DSE, cognitive impairment, and mental health, and do these associations reflect domain-specific or transdiagnostic patterns of recovery?

The same analytical approach outlined in question 1 (i.e., Pearson correlations and regression models) was conducted replacing the overall professional intervention score with its two component measures: *clinical psychological therapies* and *special educational support*.

To answer this question, we divided participants into two groups using a median split based on the sample SCQ Total score at age 11. This was available for 150 of the 165 participants (90% of the sample). Participants with scores greater than or equal to the median (≥ 3) were assigned to the high autism group (*n* = 80), while those with scores below the median (< 3) were assigned to the low autism group (*n* = 70). In our previous papers, our analytical strategy compared the 26 individuals identified with QA symptoms with the non-QA group (i.e., Rodriguez-Perez et al., 2023). The current study adopted this median split approach for two reasons. First, the QA vs. non-QA strategy severely limited statistical power for examining differential predictor effects across subgroups, particularly when examining multiple predictor domains simultaneously. While median splits involve some loss of power [59, 60], we prioritised the ability to examine how predictors work differently across meaningful subgroups, which directly addresses our research questions about outcome heterogeneity in this population. Second, while the QA phenotype represents a distinctive presentation following severe early deprivation [19], we know from previous analyses that autism symptoms in the Romanian adoptees are also elevated in deprived but non-QA adoptees, suggesting a continuum of risk [18]. The median split approach captures this continuous variation more effectively than the QA vs. non-QA approach. Following this, we repeated the analytical steps outlined in questions 1 and 1a for the two subgroups.

## Research Question 4: Are the differential effects for high vs.. low symptom levels on predictions specific to autism or do their generalise to ADHD?

To examine the issue of specificity, we applied the same median split approach using ADHD symptom severity at age 11. In this case, we used the continuous scores from the Revised Rutter Scale [58] to ensure an evened distribution of groups. In this way, symptoms of hyperactivity, sustained attention, impulsivity and distractibility were measured on a continuous scale (0–2), and then a total score, with a maximum score of 8, was calculated adding the individual scores. The median split created a low ADHD (*n* = 81; median < 2) and a high ADHD (*n* = 67; median ≥ 2) group (ADHD data was missing for 11% of participants).

All tests, except LGM, were run using SPSS version 29.0.2. For LGM, we used MPlus version 8.7.

## Results

Table 1 describes the sample characteristics at age 11 years broken down by whether they were included in the high vs. low autism groups (according to the median split cut-off). The high autism group experienced significantly longer periods of institutional deprivation and showed substantially higher autism symptom severity. They also demonstrated greater impairments in other developmental areas, including higher rates of ADHD symptoms and DSE, alongside lower IQ scores. Beyond these core neurodevelopmental differences, the high autism group experienced more adverse childhood environments, including higher exposure to negative family and peer experiences, more adverse life events and greater maternal mental health problems. They also received more *professional intervention* during childhood, with higher rates of *clinical psychological therapies* and *special educational support* relative to the low autism group.

### Preparatory analysis

#### Negative family and peer experiences

The PCA including maternal mental health, bullying victimisation, and adverse life events had sampling adequacy (KMO = 0.56), and sufficiently large correlations (χ2 (3) = 12.70, *p* <.001). One component with an eigenvalue greater than 1 explained 45.96% of the variance. A composite score was derived.

Professional intervention: The PCA including *clinical psychological therapies* and *special educational support* had adequate sampling (KMO = 0.60), and a significant sphericity test (χ2 (1) = 20.08, *p* <.001). A single component with an eigenvalue greater than 1 explained 65.08% of the variance. A composite score was subsequently derived.

Young-adult mental health: We reduced the number of variables measuring mental health difficulties in young-adulthood derived from the parent-rated CBRS scores for generalised anxiety, major depression, oppositional defiant disorder, manic episode, social phobia, conduct, and obsessive-compulsive disorder. The PCA had sampling adequacy (KMO = 0.82), and a significant Baertlett’s test of sphericity (χ2 10) = 265.92, *p* <.001). The single component had an eigenvalue greater than 1, which explained 74.21% of the variance, and was labelled *mental health in young-adulthood*.

## Question 1: Are *negative family and peer experiences* and/or *professional intervention* in childhood associated with long-term neurodevelopmental trajectories -including autism, ADHD, DSE, and cognitive impairment – as well as young-adult, mental health outcomes?

### Univariate associations

Table 2 reports the bivariate associations between predictors and outcomes in the total sample. Higher levels of *professional intervention* in childhood were significantly correlated with improving trajectories across most outcomes: specifically, symptom reduction (negative slopes) for autism, DSE, and ADHD symptoms and improving IQ trajectories (positive slopes). Contrary to expectations, greater exposure to *negative family and peer experiences* during childhood was significantly correlated with improving autism and ADHD symptom trajectories (i.e., symptom reduction – negative slope) (Table 2).

**Table 2:** Bi-variate correlations (r) between predictors and outcomes for the total sample and subgroups

		Total sample (*n* = 165)	Autism symptom groups		ADHD symptom groups	
			Low (*n* = 70)	High(*n* = 80)	Low (*n* = 81)	High (*n* = 67)
Childhood Negative Family & Peer experiences						
**Outcome trajectories**	**Autism**	− 0.17*	− 0.02	− 0.13	− 0.14	0.07
	**ADHD**	− 0.22*	− 0.32*	− 0.15	− 0.1	− 0.13
	**DSE**	− 0.01	− 0.02	0.04	− 0.11	0.14
	**IQ**	− 0.13	0.01	− 0.02	0.12	− 0.01
	**Mental health**	− 0.01	− 0.11	0.04	− 0.02	0.01
**Childhood Professional intervention – total score**						
**Outcome trajectories**	**Autism**	− 0.35**	− 0.22	− 0.30**	− 0.30**	− 0.35**
	**ADHD**	− 0.38**	− 0.24*	− 0.41**	0.18	− 0.37*
	**DSE**	− 0.30**	− 0.23	− 0.29*	− 0.46**	− 0.11
	**IQ**	0.24*	0.14	0.29*	0.11	0.36*
	**Mental health**	0.07	− 0.02	0.21	0.03	0.16
**Childhood Professional intervention – special educational support**						
**Outcome trajectories**	**Autism**	− 0.24**				
	**ADHD**	− 0.40**				
	**DSE**	− 0.22*				
	**IQ**	0.09				
	**Mental health**	0.13				
**Childhood Professional intervention – special educational support**						
**Outcome trajectories**	**Autism**	− 0.33***				
	**ADHD**	− 0.21*				
	**DSE**	− 0.25*				
	**IQ**	0.29**				
	**Mental health**	− 0.02				

High vs. low autism and ADHD symptom groups based on a median split. See Methods section for detail

*SE* Disinhibited Social Engagement. *ADHD* Attention Deficit Hyperactivity DisorderA

* = *p* <.05; ** = *p* <.01; *** = *p* <.001

### Multivariate predictions

Table 3 reports the results for the multivariate regression analyses including both *professional interventions* and *negative family and peer experiences* as predictors in the total sample. These analyses confirmed the above relationships after accounting for both experience-related predictors simultaneously along with background characteristics – *professional intervention* remained significantly associated with improving trajectories across autism, DSE and ADHD symptoms, and IQ scores. The significant univariate predictions for *negative family and peer experiences* were no longer significant in multivariate models. Against expectations, no significant associations were found between *professional intervention* or *negative family and peer experiences* and mental health outcomes in either univariate or multivariate models.

**Table 3:** Multivariate analyses of predictors and outcomes in full sample

**Full sample**		**Predictors**					
**Outcome trajectories**	**Model summary**	**Negative Family & Peer experiences**	**Childhood Professional intervention**		**Sex**	**Age**	**Duration of deprivation**
**Autism**	**F (5**,** 140) = 5.53** ***p*** ***<****.001.*$\:{\boldsymbol{R}}^{2}$**= 0.14**	− 0.08	− 0.29***		− 0.12	0.01	− 0.18*
**DSE**	**F (5**,**140) = 5.60**, ***p <.001***,$\:{\boldsymbol{R}}^{2}$**=0.14**	0.11	− 0.25**		− 0.03	− 0.01	− 0.29***
**ADHD**	**F (5**,**141) = 6.41**, ***p <.001***,$\:{\boldsymbol{R}}^{2}$**= 0.16**	− 0.13	− 0.34***		− 0.16	− 0.04	− 0.04
**IQ**	**F (5**,**140) = 6.04**, ***p <.001***,** R2 = 0.15**	− 0.10	0.19*		− 0.09	− 0.01	0.33***
**Mental Health**	**F (10**,**80) = 3.78**, ***p <.001***,$\:{\boldsymbol{R}}^{2}$**=0.24**	− 0.04	**0.10**		− 0.04	− 0.07	0.21
**Full sample**			**Childhood Professional intervention**				
**Outcome trajectories**	**Model summary**	**Negative Family & Peer experiences**	**Clinical psychological therapies**	**Special educational support**	**Sex**	**Age**	**Duration of deprivation**
**Autism**	**F (6**,** 139) = 4.82**, ***p <.001***,$\:{\boldsymbol{R}}^{2}$**= 0.14**	− 0.09	− 0.12	− 0.25**	− 0.13	0.01	− 0.16*
**DSE**	**F (6**,**139) = 6.41**, ***p <.001***,$\:{\boldsymbol{R}}^{2}$**= 0.17**	0.11	− 0.20*	− 0.12	− 0.03	− 0.01	− 0.29***
**ADHD**	**F (6**,**140) = 5.78**, ***p <.001***,$\:{\boldsymbol{R}}^{2}$**= 0.16**	− 0.11	− 0.32*	− 0.11	− 0.14	− 0.03	− 0.06
**IQ**	**F (6**,**139) = 5.38**, ***p <.001***,$\:{\boldsymbol{R}}^{2}$**=0.15**	− 0.08	0.03	0.21*	− 0.07	− 0.01	0.31***
**Mental Health**	**F (11**,**79) = 2.16**, ***p <.001***,$\:{\boldsymbol{R}}^{2}$**= 0.16**	− 0.06	0.07	0.06	− 0.07	− 0.11	0.19

Values represent standardised beta coefficients. Full regression results for Mental health outcomes can be seen Appendix II – Table 4

* *p* <.05; ** *p* <.01; *** *p* <.001. DSE; Disinhibited Social Engagement. ADHD; Attention Deficit Hyperactivity Disorder

## Question 2: What is the relative importance of clinical psychological therapies and special educational support in childhood in their association with trajectories of autism, ADHD, DSE, cognitive impairment, and mental health, and do these associations reflect domain-specific or transdiagnostic patterns of recovery?

### Univariate associations

Both clinical psychological therapies and special educational support were correlated with symptom reduction (improving trajectories) for autism, ADHD, and DSE; in addition, the latter was also correlated with improving IQ trajectories (Table 2).

### Multivariate predictions

Table 3 presents the results from the multivariate models. *Clinical psychological therapies* and *special educational support* appeared to operate in complementary ways. The former was significantly associated with improving DSE and ADHD symptom trajectories, while the latter was associated with improving autism and IQ trajectories.

## Question 3: Do the associations between childhood predictors and young-adult outcomes vary based on baseline symptom severity (comparing high- vs. low-symptom groups)?

### Univariate predictions

Table 2 reports the bivariate correlations between predictors and outcomes in groups defined by high vs. low autism symptoms. In general, *professional intervention* showed correlations of similar size for improving autism, ADHD, DSE, and IQ trajectories across both autism groups. Statistical significance was largely limited to the high autism group, with the exception of significant associations with improving ADHD trajectories in the low autism group.

### Multivariate predictions

Table 4 reports the results for the multivariate regression analyses including both predictors and background characteristics as covariates in the groups divided into high and low autism symptoms. Multivariate models revealed a more nuanced picture with similar effect sizes seen for improving autism, DSE, and IQ trajectories when *negative family* and *peer experiences* and background characteristics were controlled for. While the categorical nature of our group definitions (high vs. low) precludes a formal comparison of how predictors map onto QA vs. non-QA cases, the results suggest that the impact of early intervention is not limited to those meeting the QA threshold but extends across the broader spectrum of autism-related risk. The exception was ADHD, which showed a substantially larger effects in the high autism group. However, because of issues of statistical power, significance was largely limited to the high autism group. *Negative family and peer experiences* did not show significant relationships with any outcome trajectories.

**Table 4:** Multivariate analyses of predictors of different outcome trajectories by severity group

	Autism groups						ADHD groups					
**Outcome trajectories**	**Model**	**Predictors**					**Model**	**Predictors**				
		**Negative family & peer experiences**	**Professional intervention**	**Sex**	**Age**	**Deprivation duration**		**Negative family & peer experiences**	**Professional intervention**	**Sex**	**Age**	**Deprivation duration**
**Low symptom group (***n* **= 70)**							**Low symptom group (***n* **= 83)**					
**Autism**	F (5, 60) = 1.60, *p* =.17,$\:{R}^{2}$= 0.04	0.08	− 0.25	− 0.19	− 0.19	0.04	**F (5**,** 69) = 2.34**, ***p =.05***,$\:{\boldsymbol{R}}^{2}$**= 0.08**	− 0.02	− 0.23	− 0.09	− 0.03	− 0.23*
**DSE**	F (5,60) = 1.43, *p* =.23,$\:{R}^{2}$= 0.03	0.12	− 0.27	− 0.13	− 0.06	0.20	**F (5**,**68) = 4.39**, ***p =.002***,$\:{\boldsymbol{R}}^{2}$**= 0.19**	0.08	− 0.42***	0.05	− 0.13	− 0.11
**ADHD**	**F (5**,**60) = 3.01**, ***p =.02***,$\:{\boldsymbol{R}}^{2}$**= 0.12**	− 0.23	− 0.20	− 0.28**	− 0.09	− 0.01	F (5,69) = 0.98, *p* =.44,$\:{R}^{2}$= − 0.01	− 0.17	0.19	0.06	0.08	0.08
**IQ**	F (5,60) = 1.72, *p* =.14,$\:{R}^{2}$=0.05	− 0.01	0.15	− 0.14	− 0.05	0.28*	**F (5**,**68) = 3.33**, ***p =.01***,$\:{\boldsymbol{R}}^{2}$**= 0.14**	− 0.03	0.03	− 0.14	− 0.08	0.41***
**Mental Health**	F (10,36) = 1.08, *p* =.40,$\:{R}^{2}$= 0.02	− 0.27	− 0.06	− 0.17	− 0.22	0.28	F (10,36) = 0.86, *p* =.58,$\:{R}^{2}$= 0.03	− 0.08	0.05	0.04	− 0.12	0.13
**High symptom group (***n* **= 80)**							**High symptom group (***n* **= 67)**					
**Autism**	**F (5**,** 70) = 2.27**, ***p =.05***,$\:{\boldsymbol{R}}^{2}$**= 0.14**	− 0.10	− 0.23*	0.09	0.14	0.18	F (5, 59) = 2.35, *p* =.05,$\:{R}^{2}$= 0.10	− 0.05	− 0.33*	− 0.12	0.02	− 0.18
**DSE**	**F (5**,**69) = 3.61**, ***p =.01***,$\:{\boldsymbol{R}}^{2}$**=0.15**	0.10	− 0.20*	0.01	− 0.12	− 0.35**	**F (5**,**59) = 2.80**, ***p =.03***,$\:{\boldsymbol{R}}^{2}$**= 0.12**	0.15	− 0.08	− 0.13	− 0.08	− 0.41**
**ADHD**	**F (5**,**71) = 2.99**, ***p =.02***,$\:{\boldsymbol{R}}^{2}$**= 0.14**	− 0.09	− 0.40***	− 0.09	0.01	0.03	**F (5**,**60) = 3.11**, ***p =.02***,$\:{\boldsymbol{R}}^{2}$**=0.14**	− 0.09	− 0.35**	− 0.24*	− 0.09	− 0.10
**IQ**	**F (5**,**69) = 4.04**, ***p =.003***,$\:{\boldsymbol{R}}^{2}$**= 0.17**	− 0.10	0.22*	− 0.11	− 0.09	0.35**	**F (5**,**59) = 3.08**, ***p =.02***,$\:{\boldsymbol{R}}^{2}$**=0.14**	− 0.12	0.34**	− 0.07	− 0.01	0.24*
**Mental Health**	**F (10**,**33) = 2.16**, ***p =.04***,$\:{\boldsymbol{R}}^{2}$**=0.21**	0.05	0.31	− 0.10	− 0.08	0.10	**F (10**,**32) = 2.17**, ***p =.05***,$\:{\boldsymbol{R}}^{2}$**= 0.22**	0.01	0.28	− 0.15	− 0.01	0.39*

Values represent standardised beta coefficients. Full regression results for Mental health outcomes can be seen Appendix II – Table 4.A

* *p* <.05; ** *p* <.01; *** *p* <.001. DSE Disinhibited Social Engagement. ADHD Attention Deficit Hyperactivity DisorderA

## Question 4: Are the effects of these predictors specific to autism, or do they follow similar patterns across different DSPs, such as ADHD?

### Univariate predictions

Table 2 reports the bivariate correlations between predictors and outcomes in groups defined by high vs. low ADHD symptoms. The pattern of results was more complex than for the autism groups. *Professional intervention* was significantly associated with improving ADHD and IQ trajectories in the high group only, improving DSE trajectories in the low group only, and improving autism trajectories in both groups.

### Multivariate predictions

Table 4 reports the results for the multivariate regression analyses including both predictors in the groups divided into high and low ADHD symptoms. In general, the univariate pattern persisted, with *professional intervention* remaining significantly associated with improving ADHD, autism and IQ trajectories in the high group and improving DSE trajectories in the low group. The association with improving autism trajectories in the low symptom group remained but was no longer significant. *Negative family and peer experiences* did not show significant relationships with any outcome trajectory.

## Discussion

Our previous research found that the impact of early institutional deprivation on neurodevelopmental outcomes persists into young adulthood and is associated with significant functional impairment [17, 18]. However, the substantial heterogeneity in developmental trajectories across the cohort raised a critical question: why do some individuals show greater recovery than others? Given that deprivation-specific problems (DSPs)—including autism, ADHD, DSE, and cognitive impairment—often co-occur, the current study adopted a transdiagnostic approach to explore whether childhood environmental experiences were associated with shared or distinct recovery trajectories across these domains. Specifically, we examined the longitudinal associations between two types of childhood experiences—professional intervention (*clinical psychological interventions* and *special educational support*) and negative social experiences (*negative family and peer experiences*)—and outcomes into young-adulthood. There were a number of notable findings.

First and most strikingly, *professional intervention* delivered in childhood, comprising *clinical psychological therapies* and/or *special educational support*, was consistently associated with improving symptom trajectories across a broad range of deprivation-related outcomes, including autism, ADHD, DSE and IQ. Importantly, these effects remained after accounting for the duration of deprivation experienced. These results are interesting and encouraging, as they suggest that deprivation-related problems – shown by previous research to be highly persistent across adolescence and into adult life [17] – are, in fact, tractable to a degree, given effective intervention.

Second, the apparent benefits of professional interventions across multiple distinct neurodevelopmental domains, rather than being limited to a single diagnostic category like autism. This demonstrate their transdiagnostic relevance and suggest that they may be targeting core underlying mechanisms related to the common experience of early deprivation, which research has shown affects multiple neurodevelopmental domains [17]. Both clinical psychological therapies and special educational support showed associations with improvements across autism spectrum, ADHD and DSE symptomatology, and cognitive performance, indicating that comprehensive professional support is important for addressing the multifaceted impact of institutional deprivation. However, while our results show a significant association between childhood professional intervention and improved developmental trajectories, the observational nature of the ERA study precludes a definitive causal conclusion. Rather than proving that intervention ‘caused’ symptom reduction, these associations suggest that deprivation-related difficulties—despite their general persistence—may be more tractable or responsive to environmental support than previously thought. Identifying the specific mechanisms driving these improving trajectories remains a question for future research through more controlled designs.

Third, despite their transdiagnostic value, the context and focus of the intervention (i.e., clinical or educational) appeared important in predicting the outcomes. Crucially, when both intervention types were examined simultaneously, they demonstrated distinct patterns of association with different symptom trajectories, suggesting specificity rather than general effects of the intervention and that the two modes of intervention could be considered complementary. *Special educational support* showed stronger associations with autism and IQ trajectories, while *clinical psychological therapies* were more strongly associated with improvements in ADHD and DSE symptoms. This differential pattern may reflect the fundamental differences in how these symptoms respond to distinct intervention approaches. Autism symptoms and cognitive development may benefit particularly from schools’ structured, language-rich, socially interactive settings that provide inputs missing during early deprivation. Meanwhile, ADHD symptoms—characterised by inattention, hyperactivity, and impulsivity (DSM-5 [1]), —and DSE symptoms involving social regulation challenges [61] may be more effectively addressed through clinical psychological interventions. However, without specific information about the therapeutic approaches used, these explanations remain tentative and require further investigation.

While *professional intervention* showed broad transdiagnostic associations, it is important to note that these outcomes were not uniform across all domains. IQ trajectories showed the clearest improvement, with *special educational support* significantly associated with improving IQ trajectories. In contrast, young-adult mental health outcomes showed no significant association with childhood professional intervention, even after controlling for baseline emotional problems. Furthermore, the magnitude of associations varied between symptom domains; for instance, *clinical psychological therapies* were more robustly associated with improvements in DSE and ADHD symptoms than with autism symptoms. These differential patterns suggest that while early deprivation-related difficulties are generally tractable, the degree and nature of their responsiveness vary by developmental domain, despite a putatively shared aetiological origin (i.e., exposure to institutional deprivation).

Fourth, baseline autism symptom severity (i.e., age 11) was not an important predictor of outcome trajectories. *Professional intervention* showed benefits across both autism symptom groups, with effect sizes that were often comparable in magnitude. However, the effects reached statistical significance primarily in the high autism group across most outcomes. This pattern likely reflects several factors. First, only 27 individuals in the low autism group (*n* = 70) received any form of professional intervention, compared to 51 in the high autism group (*n* = 80) (Table 1), substantially limiting statistical power to detect significant effects in the low autism group despite meaningful associations being present. Second, individuals with higher baseline symptoms (i.e., high autism group) may have had greater potential for measurable change, making improving trajectories more detectable. Overall, these findings suggest that *professional intervention* was associated with improvements across the spectrum of autism severity, with effectiveness appearing relatively independent of baseline symptom levels. These findings also inform the debate regarding the distinction between QA and idiopathic autism. Because the associations between intervention and symptom reduction were consistent across both high- and low-symptom groups, this suggests that while the aetiology of QA (deprivation-driven) might be distinct from idiopathic autism, the mechanisms of change through environmental support operate similarly across the symptom continuum, supporting a transdiagnostic model of tractability in post-deprivation recovery.

Fifth, baseline ADHD symptom severity (age 11) showed a pattern similar to autism severity in predicting intervention response. *Professional interventions* were more consistently associated with significant improvements in the high ADHD symptom group, particularly for autism, ADHD, and IQ trajectories. The low ADHD group showed fewer significant associations, with the notable exception of DSE trajectories, where intervention effects were particularly strong. This pattern likely reflects similar factors to those observed in the autism groups, including differences in intervention levels received and statistical power. Overall, while intervention benefits were detected more consistently in children with higher baseline symptom severity, this likely reflects statistical and methodological factors rather than true differences in intervention effectiveness.

Sixth, the lack of impact of *professional intervention* in childhood on young-adult mental health outcomes is surprising, particularly given that previous research found that late-emerging mental health problems were mediated via neurodevelopmental problems [59, 60]. This pattern likely reflects the nature and focus of the interventions received. Interventions at age 11 primarily targeted neurodevelopmental difficulties rather than mental health symptoms, social-emotional functioning, or peer relationships [27]. While these interventions may have reduced ADHD and autism symptoms, as per our results, they did not include components specifically designed to address emerging social vulnerabilities, peer integration, or the prevention of bullying victimisation—factors that may be critical for long-term mental health outcomes in this population [62, 63]. Despite the established mediation pathway from neurodevelopmental problems to mental health [59, 60], improving neurodevelopmental difficulties alone did not produce knock-on benefits for mental health. These findings suggest that comprehensive support for institutionally deprived populations requires not only neurodevelopmental intervention but also targeted efforts to address social-emotional development, peer relationships, and protective factors against bullying—components that were not part of the ‘generic’ clinical and educational support measured in this study.

Seventh, *negative family and peer experiences* in childhood (including peer bullying, maternal mental health problems, and adverse life events) did not emerge as significant direct predictors of developmental trajectories in our models. However, this null finding requires careful interpretation in light of recent evidence from this same cohort. Rizeq et al. [36] demonstrated that bullying victimisation played a crucial mediating role in the pathway from deprivation-related neurodevelopmental problems to adult internalising symptoms. Several methodological factors may explain why our study did not detect these effects. First, our composite measure of *negative family and peer experiences* combined multiple distinct constructs, which may have diluted the specific effects of peer victimisation documented by Rizeq et al. [36] when bullying was examined as a distinct construct. Second, our analytical approach tested direct predictive effects on trajectories, whereas Rizeq et al.‘s cross-lagged mediation models captured indirect cascading pathways (deprivation → neurodevelopmental problems → bullying → mental health) that may not be detectable when testing only direct effects. These findings highlight important methodological considerations: composite measures of negative experiences may obscure specific risk pathways, and the distinction between direct and mediated effects is crucial for understanding how early adversity shapes long-term outcomes in institutionally deprived populations.

On the face of it, these findings are in line with extensive general literature supporting the value of early and ongoing interventions for neurodevelopmental conditions [29, 30], extending this body of literature to a population whose difficulties arise following their exposure to extreme institutional deprivation – a group whose problems may be particularly persistent across adolescence into young-adulthood [18, 61, 64]. Crucially, the benefits were seen across a broad range of outcomes, including core DSPs, which suggests that deprivation-related neurodevelopmental difficulties remain responsive to professional interventions. This challenges the notion that, once established, institutional deprivation sets in motion developmental processes that are unalterable, and instead suggests that, with appropriate support, these children can experience at least a certain level of improvement. Similar to intervention studies for idiopathic conditions, our findings show domain-specific effects, with different intervention types showing associations with different neurodevelopmental domains, consistent with reports for idiopathic autism [32–34] and ADHD [65, 66]. However, direct comparative studies examining intervention effectiveness between deprivation-related and idiopathic neurodevelopmental conditions are needed to determine whether etiological differences influence intervention responsiveness and long-term outcomes.

The current study has some limitations. First, developmental trajectories estimated from three time points using linear models may have missed complex, non-linear patterns. However, the consistency of findings using this linear approach for some predictors (e.g., the strong associations with professional intervention) and the subgroup analyses suggest that these models were able to capture meaningful developmental processes. Second, assessing predictors only at age 11 may have missed earlier or later predictive effects. However, this timing captures functioning after initial post-adoption catch-up [67] and before major adolescent transitions (e.g., the significant change to secondary education), representing a particularly relevant developmental window [68]. Third, treating DSPs trajectories as separate outcomes despite considerable overlap between autism, ADHD, DSE and cognitive impairment [17] may have missed an element of shared underlying vulnerability that manifests in different DSPs. Conducting the additional ADHD subgroup analyses may have partially addressed this issue. Fourth, we lacked specific information about the therapeutic approaches used within our clinical psychological therapies measure, which limits our ability to inform clinical practice on which interventions were most effective. Fifth, this study used an observational design rather than a Randomised Controlled Trial (RCT), which limits causal inferences about the effectiveness of the intervention. However, RCTs involving institutionally deprived populations are neither ethical nor feasible, as they would require deliberately not providing potentially beneficial interventions to a vulnerable population. While the current observational design cannot establish causality, it represents the most rigorous approach possible for studying intervention effects in this population, particularly given the natural experiment provided by the ERA cohort. Finally, the sample size limited the statistical power to detect relatively small associations, especially in the sub-group analysis.

Our findings have implications for practitioners and service providers. First, though using an observational design, our results suggest that professional intervention during childhood may be important in improving long-term neurodevelopmental outcomes in institutionally deprived children. Second, clinical and educational services appear to play complementary roles in addressing the needs of children with a history of institutional deprivation. Third, while interventions targeting neurodevelopmental problems show benefits for core symptoms, they do not appear sufficient to prevent later mental health difficulties. This suggests that comprehensive support must also include specific attention to social-emotional development, peer relationships, and bullying prevention—factors that may independently drive mental health outcomes even when neurodevelopmental symptoms improve.

In summary, childhood *professional intervention* was associated with improved deprivation-specific neurodevelopmental outcomes in the ERA cohort, with effects observed across symptom severity levels, highlighting the tractable nature of these problems, despite their strong persistence across development. The differential and complementary effects of educational and clinical interventions across different neurodevelopmental domains suggest that comprehensive support requires access to both intervention modalities.

## Supplementary Information

Below is the link to the electronic supplementary material.

Supplementary Material 1

## Data Availability

No datasets were generated or analysed during the current study.
