# Supplementary material for: Childhood post-adoption experiences and the long-term trajectories of deprivation-specific neurodevelopmental problems: a longitudinal study of the English and Romanian adoptee cohort
Source: Eur Child Adolesc Psychiatry. 2026 Feb 27;35(6):1867–82. doi: 10.1007/s00787-026-02968-x (PMC13337662; doi:10.1007/s00787-026-02968-x)
Supplement: Supplementary file 1 — Supplementary Material 1 [file 787_2026_2968_MOESM1_ESM.docx]

**Childhood Post-Adoption Experiences and the Long-term Trajectories of Deprivation-Specific Neurodevelopmental Problems: A Longitudinal Study of the English and Romanian Adoptee Cohort.**

Maria Rodriguez Perez, Mark Kennedy, Jana Kreppner, & Edmund J.S. Sonuga-Barke

**Contents**

I: Selection of Social Communication Questionnaire (SCQ) items

II: Supplementary statistical analyses

**I: Items used for autism symptoms domains across age waves**

Preamble: To ensure its developmental appropriateness in young adulthood, items from the full SCQ were dropped on the basis of their distribution in the combined UK and Rom<6 group because they were (i) too commonly endorsed in young adulthood and/or (ii) showed a substantial increase between age 6 years and young adulthood - patterns inconsistent with items being considered markers of a serious/rare neuro-developmental condition. Full item descriptions are withheld due to copyright.

**Table 1: Items by autism symptom domain by waves**

| ***Autism Spectrum Disorder*** |  |  |  |
| --- | --- | --- | --- |
| **Age 6** | **Age 11** | **Age 15** | **YA - Parent report** |
| **Social Reciprocal Interaction** | As age 6 | As age 6 | As age 6 |
| 17. Smiles back |  |  |  |
| 21. Attempts to comfort |  |  |  |
| 23. Normal range of facial expressions |  |  |  |
| 24. Appropriate facial expressions |  |  |  |
| 28. Responds positively to others |  |  |  |
| **Communication** |  |  |  |
| 9. Odd speech |  |  |  |
| 10. To-and-fro conversation |  |  |  |
| 11. Socially appropriate |  |  |  |
| 12. Difficulties with pronouns |  |  |  |
| 13. Uses made up words/phrases |  |  |  |
| **Repetitive and Stereotyped Behaviours** |  |  |  |
| 33. Odds interest |  |  |  |
| 34. Interested in parts of objects. |  |  |  |
| 35. Ritualised behaviour |  |  |  |
| 36. Unusual interest in smell etc. of things or people |  |  |  |
| 38. Odd mannerisms/movement |  |  |  |

*Note*. Item description from the SCQ is withheld due to copyright. YA, Young adulthood.

**References:**

Rutter M, Bailey A, Lord C. SCQ. The social communication questionnaire. Torrance: Western Psychological Services, 2003

**Table 2: Excluded items and reason for exclusion**

| **Item** | **Commonly endorsed in young adulthood** | **Increased between age 6 and young adulthood** |
| --- | --- | --- |
| 4. Spontaneously point at things | X | X |
| 6. Nod head for yes | X | X |
| 7.Shake head for no | X | X |
| 8. Talk to be friendly | X |  |
| 14. Repetitive speech | X |  |
| 16. Eye contact | X |  |
| 18. Show objects of interest | X |  |
| 19. Share interest | X | X |
| 20. Wants others to join in | X | X |
| 22. Speech and gestures to get attention | X |  |
| 26. Plays make believe games | X | X |
| 27. Interest in peers |  |  |
| 29. Plays imaginative games | X | X |
| 30. Special interests | X |  |
| 31. Joins in with games | X |  |
| 32. Any particular friends | X |  |
| 39. Stereotyped behaviours | X |  |

*Note*. The following items were not included as they do not constitute part of any subscale; 15. Attends to you, 37. Special objects, 40. Selfharm/ head banging.

**II: Supplementary statistical analyses**

**Table 3: Correlations (r) between individual predictor variables within each of the domains**

| ***Negative family and peer experiences*** | **Adverse Life events** | **Maternal mental health** | **Bullying victimization** |
| --- | --- | --- | --- |
| Adverse Life events | — |  |  |
| Maternal mental health | **.21**** | — |  |
|  | (115) | (140) |  |
| Bullying victimization | **.30***** | 0.08 | — |
|  | (106) | (118) | (132) |
|  |  |  |  |
| **Professional intervention** | **Clinical care** | **Educational support** |  |
| Clinical care | — |  |  |
| Educational support | **.26***** | — |  |
|  | (161) | (161) |  |

Note. Numbers in parentheses represent sample sizes. ***** **p < .05**; ****** **p < .01**; ******* **p < .001**.

**Table 4: Multivariate analyses of predictors and mental health outcome by group**

| **Predictors** | **Mental health outcome** | **Full sample** | **Low Autism** | **High Autism** | **Low ADHD** | **High ADHD** |
| --- | --- | --- | --- | --- | --- | --- |
|  | **Model summary** | **F (11,79) = 3.26, p = .001,** $\boldsymbol{R}^{\mathbf{2}}$ **= .20** | F (10,36) = 1.08, p = .40, $R^{2}$ = -.02 | **F (10,33) = 2.16, p = .04,** $\boldsymbol{R}^{\mathbf{2}}$ **= .21** | F (10,36) = 0.86, p = .58, $R^{2}$ = 0.03 | **F (10,32) = 2.17, p = .05,** $\boldsymbol{R}^{\mathbf{2}}$ **= 0.22** |
|  | **Negative Family & Peer experiences** | -.04 | -.27 | .05 | -.08 | .01 |
|  | **Professional intervention** | 0.1 | -.06 | .31 | .05 | .28 |
|  | **Sex** | -.04 | -.17 | -.10 | .04 | -.15 |
|  | **Age** | -.07 | -.22 | -.09 | -.12 | -.01 |
|  | **Duration of deprivation** | .**21** | .28 | .10 | .13 | .**39*** |
|  | **Emotional problems 11** | .19 | .16 | .28 | .27 | .18 |
|  | **Autism age 11** | **.25*** | .29 | -.01 | .30 | .05 |
|  | **ADHD age 11** | .17 | **.39*** | .05 | .02 | -.03 |
|  | **DSE age 11** | .04 | -.15 | .19 | -.01 | .03 |
|  | **IQ age 11** | .06 | -.02 | .08 | .13 | .06 |

Note: Values represent standardised beta coefficients.

* p < .05; ** p < .01; *** p < .001. DSE; Disinhibited Social Engagement. ADHD; Attention Deficit Hyperactivity Disorder
